# Downregulation of a novel flagellar synthesis regulator AsiR promotes intracellular replication and systemic pathogenicity of *Salmonella* Typhimurium

**DOI:** 10.1080/21505594.2020.1870331

**Published:** 2021-01-07

**Authors:** Shuangshuang Ma, Lingyan Jiang, Jingting Wang, Xiaoqian Liu, Wanwu Li, Shuai Ma, Lu Feng

**Affiliations:** a The Key Laboratory of Molecular Microbiology and Technology, Ministry of Education, Nankai University, Tianjin, China;; b TEDA Institute of Biological Sciences and Biotechnology, Tianjin Key Laboratory of Microbial Functional Genomics, Nankai University, Tianjin, China

**Keywords:** *Salmonella* Typhimurium, transcriptional regulator, asir, flagellar gene expression, acidic pH

## Abstract

The intracellular pathogen *Salmonella enterica* serovar Typhimurium (*S*. Typhimurium) exploits host macrophage as a crucial survival and replicative niche. To minimize host immune response stimulated by flagellin, the expression of flagellar genes is downregulated during *S*. Typhimurium growth within host macrophages. However, the underlying mechanisms are largely unknown. In this study, we show that STM14_1285 (named AsiR), a putative RpiR-family transcriptional regulator, which is downregulated within macrophages as previously reported and also confirmed here, positively regulates the expression of flagellar genes by directly binding to the promoter of *flhDC*. By generating an *asiR* mutant strain and a strain that persistently expresses *asiR* gene within macrophages, we confirmed that the downregulation of *asiR* contributes positively to *S*. Typhimurium replication in macrophages and systemic infection in mice, which could be attributed to decreased flagellar gene expression and therefore reduced flagellin-stimulated secretion of pro-inflammatory cytokines IL-1β and TNF-α. Furthermore, the acidic pH in macrophages is identified as a signal for the downregulation of *asiR* and therefore flagellar genes. Collectively, our results reveal a novel acidic pH signal-mediated regulatory pathway that is utilized by *S*. Typhimurium to promote intracellular replication and systemic pathogenesis by repressing flagellar gene expression.

## Introduction

*Salmonella* is recognized as a major foodborne pathogen, causing self-limiting gastroenteritis or life-threatening systemic disease in both humans and a broad range of animals[1]. Infection by *Salmonella* represents a considerable burden worldwide, with more than 90 million human cases of gastroenteritis and 20 million human cases of systemic disease being reported each year [2,3]. Of the 2600+ serovars identified so far, *Salmonella enterica* serovar Typhimurium (*S*. Typhimurium) represents a primary pathogenic serovar[4]. Infection by *S*. Typhimurium results in gastroenteritis in humans but leads to a typhoid-like systemic infection in mice[5]. The mouse model of *S*. Typhimurium has been extensively used to investigate the human systemic disease and the mechanisms of *Salmonella* virulence[6].

*Salmonella* infection generally begins with the consumption of contaminated water or food[7]. After it travels along the host’s gastrointestinal tract, *Salmonella* invades the intestinal epithelium and ultimately spreads to the liver and spleen to induce systemic infection[8]. Invasion of intestine epithelial cells and replication inside host macrophages are two hallmarks of *Salmonella* pathogenesis[1], which are necessary for gastroenteritis and systemic disease, respectively.

During the intracellular phase, *Salmonella* resides and replicates within a unique vacuolar compartment termed *Salmonella*-containing vacuole (SCV)[9]. *Salmonella* encounters several stresses in the SCV, including low magnesium (Mg^2+^) and phosphate (P_i_) concentration, mildly acidic pH, and reactive oxygen species[10]. To resist these intracellular antibacterial factors, *Salmonella* species have developed a variety of virulence regulatory mechanisms [11,12]. Several regulatory proteins have been previously identified to be involved in the regulatory network and have been reported to contribute to *Salmonella* intracellular replication in response to various environmental stimuli. PagR, a LacI family transcriptional regulator, is induced by the low Mg^2+^ and low P_i_ stimuli in host macrophages, and it activates the expression of *Salmonella* pathogenicity island (SPI)-2 genes, which are essential for *Salmonella* intracellular replication[13]. The two-component regulatory systems, PhoP/Q and EnvZ/OmpR, also activate the expression of SPI-2 genes in response to low Mg^2+^, acidic pH, and the presence of antibacterial peptides inside host macrophages (PhoP/Q)[14] or acidic pH (EnvZ/OmpR)[15]. The putative LysR-type transcriptional regulator Hrg was found to repress the *uvrA* and *katG* genes to reduce ROS production in macrophages and thus favor intracellular survival of *Salmonella* [16,17].

The flagella contribute to *S*. Typhimurium pathogenesis by promoting bacterial adherence to and invasion of host epithelial cells, providing a survival advantage during the early stages of *S*. Typhimurium infection [18,19]. However, during the systemic phase of infection, flagellin can be easily detected by the host immune system, which triggers an elevated innate immune response, including secretion of IL-1β and TNF-α, resulting in bacterial clearance from the host[20]. Flagella overexpression in *S*. Typhimurium leads to a reduction of bacterial growth in murine macrophages and to attenuation of bacterial virulence in mice[21]. The transcriptome and proteome landscape of intracellular *S*. Typhimurium has revealed that the expression of most flagellar genes is significantly downregulated after the entry of *S*. Typhimurium into murine macrophages [22–24]. The deletion of *STM1697*, which encodes an EAL-like protein, resulted in the upregulation of flagellar gene expression, leading to the attenuated ability of *S*. Typhimurium to colonize the host organs[25]. These results implicate that the downregulation of flagellar genes in macrophages contributes to the successful intracellular replication and full virulence of *S*. Typhimurium. Several studies have focused on the molecular mechanisms associated with the downregulation of flagellar genes in response to the stimuli within macrophages. Flagellar gene expression can be repressed by PhoP/Q in response to acidic pH[26]. A leader mRNA originated from the *mgtCBR* virulence operon, which is highly induced by low Mg^2+^, binds to the coding region of the flagellin gene *fljB* to induce *fljB* mRNA degradation and thus repress flagellin gene expression[27].

STM14_1285, herein named AsiR (Acid signal-induced regulator), is predicted to be an RpiR family transcriptional regulator in *S*. Typhimurium and comprises a predicted DNA˗binding helix˗turn˗helix (HTH) motif at its N terminus (https://blast.ncbi.nlm.nih.gov/Blast.cgi). The regulatory function of AsiR in *S*. Typhimurium is currently unknown. The regulatory functions of RpiR-family regulators are mostly investigated in *Lactococcus lactis* and are generally known to be involved in the regulation of carbon metabolism[28]. However, IdoR, an RpiR-family regulator in *S*. Typhimurium, has been shown to repress the expression of SPI-2 effector gene *srfJ*[29], implying that members in this family also have other functions including the regulation of virulence genes.

The expression of *asiR* is downregulated after *S*. Typhimurium entry into murine macrophages[10], but its implication in *S*. Typhimurium virulence remains elusive. Here, we demonstrated that the downregulation of *asiR* expression facilitates *S*. Typhimurium replication in murine macrophages and systemic pathogenesis in mice. Further investigation revealed that AsiR positively regulates the expression of flagellar genes and that the downregulation of AsiR leads to a decrease in flagellar gene expression and pro-inflammatory cytokine secretion. Finally, we found that the mildly acidic pH in macrophages is a trigger for the downregulation of *asiR*.

## Materials and methods

### Bacterial strains, plasmids, and growth conditions

*S*. Typhimurium strain 14028s (wild-type, WT) and its derivative strains, as well as plasmids used in this study, are described in Table 1. Primers used are described in Supplementary Table 1. Bacteria were routinely cultured in conventional Luria-Bertani (LB) medium containing tryptone 10 g·L ^−1^, yeast extract 5 g·L ^−1^, and NaCl 10 g·L ^−1^. For quantitative real-time PCR (qRT-PCR) experiments, overnight bacterial cultures in LB were diluted at 1:100 into fresh LB or N-minimal medium[30] and then incubated in a shaking incubator (37°C, 180 rpm) to reach stationary phase. Antibiotics were added into the culture medium to achieve the following final concentrations: kanamycin (Km), 50; chloramphenicol (Cm), 25; blasticidin (Bs), 100; gentamicin (Gm): 20 or 100 μg·mL ^−1^.

**Table 1.** Plasmids and bacterial strains used in this study

Plasmid or strain	Genotype or description	Source
**Plasmids**		
pKD3	For λ Red recombination; Cm^R^	Lab collection
pKD4	For λ Red recombination; Km^R^	Lab collection
pSim17	For generating mutant strains with λ Red recombinase system; Bs^R^	Lab collection
PCP20	Temperature-sensitive replicon expressing the FLP gene to remove antibiotic resistance of mutant strains; Ap^R^	Lab collection
pBluescript II KS(+)	A cloning vector; Ap^R^	Lab collection
pMS402	For construct promoter-*lux*CDABE reporter fusion; Km^R^	Lab collection
pET28a	T_7_ Expression vector; Km^R^	Lab collection
P-*asiR*	pBluescript plasmid carrying the WT *asiR* gene; Ap^R^	This study
P-*flhDC*	pBluescript plasmid carrying the WT *flhDC* gene; Ap^R^	This study
P-*asiR-lux*	pMS402 containing *asiR* promoter region cloned between *Xho*I and *BamH*I sites; Km^R^	This study
P-*ssrA-lux*	pMS402 containing *ssrA* promoter region cloned between *Xho*I and *BamH*I sites; Km^R^	This study
P-*flhDC-lux*	pMS402 containing *flhDC* promoter region cloned between *Xho*I and *BamH*I sites; Km^R^	This study
pET-*asiR*	pET28a carrying the WT *asiR* gene; Km^R^	This study
***S*. Typhimurium strains**		
WT	Wild-type *S*. Typhimurium strain 14028s	Lab collection
Δ*asiR*	WT strain *asiR*::Cm; Cm^R^	This study
*asiR* ^on^	WT strain *asiR* promoter replaced by *ssrA* promoter; Cm^R^	This study
Δ*flhDC*	WT strain *flhDC*::Km; Km^R^	This study
Δ*asiR*Δ*flhDC*	WT strain *asiR*::Cm, *flhDC*::Km; Cm^R^, Km^R^	This study
cAsiR	Δ*asiR* containing plasmid P-*asiR*; Cm^R^, Ap^R^	This study
Δ*asiR*Δ*flhDC+*p-*asiR*	Δ*asiR*Δ*flhDC* containing plasmid P-*asiR*; Cm^R^, Km^R^, Ap^R^	This study
Δ*asiR*Δ*flhDC+*p-*flhDC*	Δ*asiR*Δ*flhDC* containing plasmid P-*flhDC*; Cm^R^, Km^R^, Ap^R^	This study
WT+p-*asiR-lux*	WT containing plasmid P-*asiR-lux*; Km^R^	This study
WT+p-*ssrA-lux*	WT containing plasmid P-*ssrA-lux*; Km^R^	This study
WT+p-*flhDC-lux*	WT containing plasmid P-*flhDC-lux*; Km^R^	This study
Δ*asiR* +p-*flhDC-lux*	Δ*asiR* containing plasmid P-*flhDC-lux*; Cm^R^, Km^R^	This study
*asiR* ^on^ +p-*flhDC-lux*	*asiR* ^on^ containing plasmid P-*flhDC-lux*; Cm^R^, Km^R^	This study
cAsiR +p-*flhDC-lux*	cAsiR containing plasmid P-*flhDC-lux*; Cm^R^, Ap^R^, Km^R^	This study

### Construction of plasmids and strains

The mutant strains (Δ*asiR, ∆flhDC,* and *∆asiR∆flhDC*) were constructed with the λ Red recombinase system[31]. The transcriptional fusions *asiR-lux, ssrA-lux,* and *flhDC-lux* were generated as described previously[13]. The amplification products of respective promoter regions were digested and cloned into the *Xho*I-*BamH*I site upstream of the *lux* genes in the plasmid pMS402. To generate a complemented strain, the amplification products (including the ORF and the upstream promoter sequence) of the corresponding genes were digested and cloned into the *EcoR*I-*BamH*I site of plasmid pBluescript and transformed the recombinant plasmids to the mutant strains.

To generate the strain *asiR*^on^ (a strain persistently expresses *asiR* gene within macrophages), we replaced the promoter of *asiR* with the promoter of the SPI-2-encoded regulator gene *ssrA* by homologous recombination. First, the promoter region of *ssrA* was amplified from the genomic DNA of WT with primers *ssrA*-promoter-F and *ssrA*-promoter-R. The chloramphenicol resistance gene was amplified from the plasmid pKD3 with primers Cm-F and Cm-R. Subsequently, the above two PCR products were purified and mixed for the overlap PCR splicing with primers *ssrA*-promoter-F and Cm-R. The resulting spliced PCR products were electroporated into WT harboring the plasmid pSim17. The promoter replaced bacteria were selected by their resistance to Cm, and verified by PCR amplification and sequencing.

To generate a construct expressing AsiR protein, the *asiR* gene was amplified from the genomic DNA of *S*. Typhimurium WT and inserted into the *EcoR*I and *BamH*I restriction sites of plasmid pET-28a to generate the plasmid pET-*asiR*. The pET-*asiR* plasmid was then transformed into *Escherichia coli* BL21 (DE3) cells to generate strain BL21+ pET-*asiR*, for the expression and purification of 6× His-tagged AsiR protein (his_6_-AsiR).

### Cell culture

The RAW264.7 macrophage cell line was obtained from the Shanghai Institute of Biochemistry and Cell Biology of the Chinese Academy of Sciences (Shanghai, China). Cells were cultured routinely in RPMI-1640 medium (Gibco) containing 10% fetal bovine serum (Gibco) and were incubated at 37°C in a humidified atmosphere containing 5% CO_2_/95% air. Cells were seeded into sterile 12-well tissue culture plates at a concentration of 1 × 10^5^ cells/well and maintained as differentiated monolayers for 48 h before infection.

### Macrophage replication assays

Macrophage replication assays were performed as previously described[30]. Briefly, *S*. Typhimurium strains (WT, Δ*asiR, asiR*^on^, and cAsiR) were incubated in LB medium overnight at 37°C with shaking (180 rpm) for 16 h. The overnight bacterial cultures were diluted and opsonized for 20 min in 10% normal mice serum (Gibco). The bacteria were added to RAW264.7 cells at a multiplicity of infection (MOI) of 10. After incubated for 45 min, the infected cells were washed with PBS for three times. Then, the cells were incubated in fresh RPMI-1640 medium supplemented with 100 μg·mL ^−1^ Gentamycin for 2 h followed by incubation in RPMI-1640 supplemented with 20 μg·mL ^−1^ Gentamycin for another 14 h. At 2 and 16 h postinfection (hpi), the intracellular bacteria were collected, serially diluted in PBS, and then spread onto LB agar for counting of bacterial colony-forming units (CFUs). Fold intracellular replication was calculated as a ratio of the intracellular CFUs at 16 hpi relative to that at 2 hpi.

### Immunofluorescence microscopy

Immunofluorescence was performed as previously described[13]. Briefly, at 2 and 16 h postinfection (hpi), the infected RAW264.7 cells were washed twice with PBS, fixed with 4% paraformaldehyde for 10 min, permeabilized with 0.1% Triton X-100 for 5 min, and then blocked with 5% BSA for 1 h. The intracellular *S*. Typhimurium cells were stained using the mouse raised monoclonal anti-*S*. Typhimurium LPS antibody (1:100 dilution, Abcam) and FITC-conjugated goat anti-mouse IgG antibody (1:200 dilution, Abcam). Nuclei were stained with DAPI (4′,6-diamidino-2-phenylindole, Invitrogen). Laser scanning confocal microscopy (Zeiss LSM800) was used to obtain the cells images and the images were analyzed with Zen 2.0 software.

### Mouse infection

All animal experiments performed here were approved by the Institutional Animal Care Committee at Nankai University (Tianjin, China). BALB/c mice (female, 6–8 weeks old) were obtained from Beijing Vital River Laboratory Animal Technology Co. Ltd (Beijing, China). In order to determine the survival rate, the mice were inoculated intraperitoneally (i.p.) with 2 × 10^4^ CFUs of WT (n = 14), Δ*asiR* (n = 15), *asiR* ^on^ (n = 14) and cAsiR (n = 14) and were monitored and recorded daily for a period of 20 days. To enumerate the bacterial burdens in mice organs, four groups of mice (n = 7/group) were inoculated i.p. with 5 × 10^4^ CFUs of WT, Δ*asiR, asiR*^on^, or cAsiR, respectively, and were euthanized after 3 days. The collected liver and spleen were weighed, homogenized and diluted in PBS, and spread onto LB agar without or with appropriate antibiotics for enumeration of CFUs. For competitive infection assays, six mice were co-infected i.p. with a 1:1 mix totaling ~5 × 10^4^ CFUs of *∆flhDC* and *∆asiR∆flhDC* and euthanized on day 3 postinfection to enumerate the bacterial burden in the liver and spleen. The competitive index (CI) was calculated as follows: (single mutant strain CFUs recovered/*∆asiR∆flhDC* CFUs recovered)/(single mutant strain CFUs inoculated/*∆asiR∆flhDC* CFUs inoculated).

## RNA preparation

The protocol of RNA preparation was conducted as previously described[13]. To obtain the RNA of intracellular *S*. Typhimurium for qRT-PCR assays, we performed the macrophage replication assays as described above. The infected macrophages were lysed on ice for 30 min in 0.1% SDS, 1% acidic phenol, and 19% ethanol in water. The cell lysates were centrifugated to isolate and harvest the intracellular bacteria. The harvested bacteria were immediately frozen in liquid nitrogen and late used to extract and purificate the total RNA.

### qRT-PCR

The RN43-EASY spin Plus Rapid Extraction Kit (Aidlab) was used for the extraction and purification of total RNA, following the manufacturer’s instructions. cDNA synthesis was performed with the PrimeScript RT Reagent kit (TaKaRa). qRT-PCR reaction was carried out in the QuantStudio 5 Real-Time PCR system (Applied Biosystems). The *16S rRNA* was used for normalization in gene expression analysis. The 2^–ΔΔCt^ method was used to calculate the fold change in tested gene expression[32].

### Motility assays

The protocol of motility assays was conducted as previously described[33]. Overnight cultures of WT and Δ*asiR* were adjusted to an OD_600_ of 1.0. One microliter of each adjusted strain was spotted on 0.3% LB agar medium. After incubation at 37°C for 8 h, the motility of the bacteria was observed by measuring the diameter of the swimming zone surrounding the inoculation site.

### Electrophoretic mobility shift assay (EMSA)

The his_6_-AsiR protein was purified as previously described[34]. The DNA fragments of *flhDC* promoter were amplified from the genomic DNA of the WT. Purified DNA fragments (40 ng) were pre-incubated with increasing quantities of purified proteins in each 20 μL binding buffer (Tris-HCl pH 7.5, 20 mM; KCl, 80 mM; MgCl_2_, 10 mM; ethylenediaminetetraacetic acid, 1 mM; bovine serum albumin, 0.1 mg·L^−1^; glycerol, 100 mL·L^−1^) for 30 min at 37°C. The reaction mixtures were resolved on native 6% polyacrylamide gels in 0.5× Tris-borate-EDTA buffer (TBE). The gels were stained with Gel Red (1:10,000 dilution in TBE) for 10 min and then observed using a UV transilluminator (Tanon).

### Enzyme-linked immunosorbent assay (ELISA)

RAW24.7 cells were infected with WT, Δ*asiR*, and *asiR*^on^ as described above. The culture supernatants were collected at 16 hpi, centrifuged, aliquoted, and stored at −80°C. The amounts of IL-1β and TNF-α secreted into the supernatant were measured using commercial ELISA kits, Mouse IL-1β Quantikine ELISA Kit (R&D Systems, MLB00C) and Mouse TNF-α Quantikine ELISA Kit (R&D Systems, MTA00), following the manufacturer’s instructions.

### Statistical analysis

All data in this study were representative of three separate experiments unless otherwise stated and displayed as mean ± SD. GraphPad Prism v7.0 software was used for the statistical analysis. Student’s unpaired t-test, one-way ANOVA test, Log-rank (Mantel-Cox) test, and Mann Whitney U-test were performed to assess the statistical significance. *P*-values of less than 0.05 were considered statistically significant.

## Results

### asiR transcription is downregulated in murine macrophages

**Figure 1.**
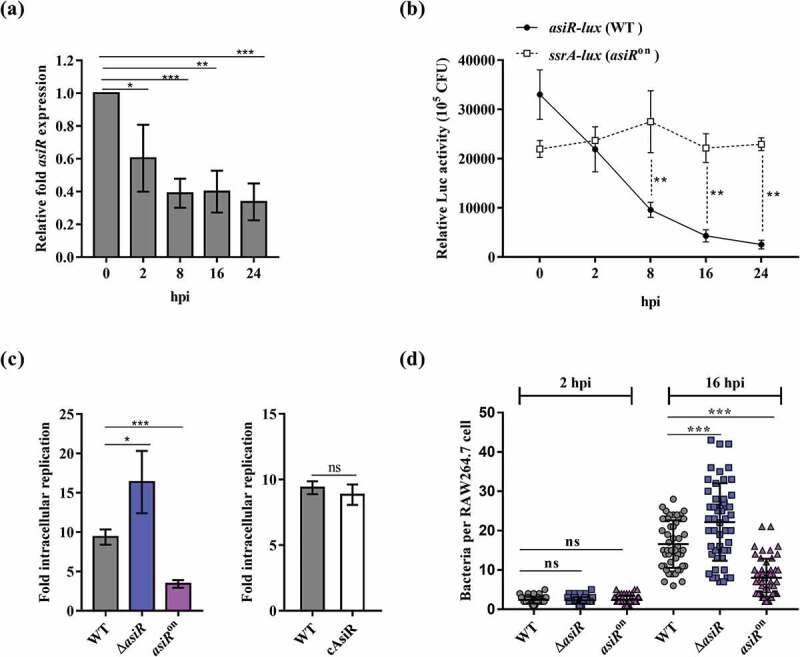
Downregulation of *asiR* enhances *S*. Typhimurium replication within macrophages. (a) qRT-PCR analysis showed the expression levels of *asiR* in RPMI-1640 medium (0 h) and in RAW264.7 cells at 2, 8, 16, and 24 h postinfection (hpi). (b) Expression of the *asiR-lux* and *ssrA-lux* transcriptional fusions was analyzed in WT growth in RPMI-1640 medium (0 h) and inside RAW264.7 cells at 2, 8, 16, and 24 hpi. (c) The replication abilities of WT, Δ*asiR, asiR*^on^ or cAsiR in RAW264.7 cells. The fold intracellular replication was calculated as a ratio of the intracellular CFUs at 16 hpi relative to that at 2 hpi. (d) Bacterial number of *S*. Typhimurium observed in per infected RAW264.7 cell (n = 50 cells) at 2 and 16 hpi. Data were obtained from three separate experiments and analyzed using Student’s t-test. *P*-values: *, *P*< 0.05; **, *P*< 0.01; ***, *P*< 0.001

To verify whether the expression of *asiR* was downregulated in host macrophages, as indicated by previously published RNA-seq data[10], murine RAW264.7 cells were infected with *S*. Typhimurium WT for a period of 24 h, and the expression of *asiR* was determined. In comparison with the bacteria cultured in RPMI-1640 medium, the expression of *asiR* was significantly decreased at 2, 8, 16, and 24 hpi, as revealed by both qRT-PCR and the bioluminescent reporter assays using an *asiR* promoter-*lux* fusion (*asiR-lux*) (Figure 1(a, b)), confirming the downregulation of *asiR* in host macrophages.

### asiR contributes negatively to S. Typhimurium replication in murine macrophages

To explore the implication of *asiR* downregulation in *S*. Typhimurium growth within macrophages, we constructed an *asiR* gene knock-out strain (Δ*asiR*), a complemented strain (cAsiR), and a strain that expresses *asiR* persistently during its growth in RAW264.7 cells (*asiR*^on^). We confirmed that the expression of *asiR* was upregulated in *asiR*^on^ within RAW264.7 cells, in contrast to the downregulation of *asiR* expression in WT, and was maintained above the WT level during the 24 h infection period (Figure 1(b) and Figure S1). Both Δ*asiR* and *asiR*^on^ grew equally well as the WT in LB and RPMI-1640 medium (Figure S2(a) and Figure S2(b)). However, when we determined the growth of those strains in RAW264.7 cells, it was found that the replication rate of Δ*asiR* was significantly increased compared to that of WT at 16 hpi and reduced to the WT level by complementation with *asiR* (cAsiR) (Figure 1(c)). On the contrary, the replication rate of *asiR*^on^ in RAW264.7 cells was significantly decreased compared to that of WT (Figure 1(c)). We further used immunofluorescence microscopy to quantify the number of intracellular *S*. Typhimurium per RAW264.7 cell. At 2 hpi, the average number of WT, Δ*asiR,* and *asiR*^on^ cells per RAW264.7 cell was similar, with one to four bacteria per RAW264.7 cell (Figure 1(d)). However, at 16 hpi, the average number of Δ*asiR* cells per RAW264.7 cell (~22 bacteria/macrophage) was significantly higher than that of WT (~16 bacteria/macrophage), while in *asiR*^on^ the number was significantly lower than that of WT (~8 bacteria/macrophage) (Figure 1(d)). These results indicate that AsiR contributes negatively to the growth of *S*. Typhimurium in host macrophages, and thus, downregulation of AsiR promotes the intracellular growth of *S*. Typhimurium.

### asiR contributes negatively to S. Typhimurium virulence in mice

**Figure 2.**
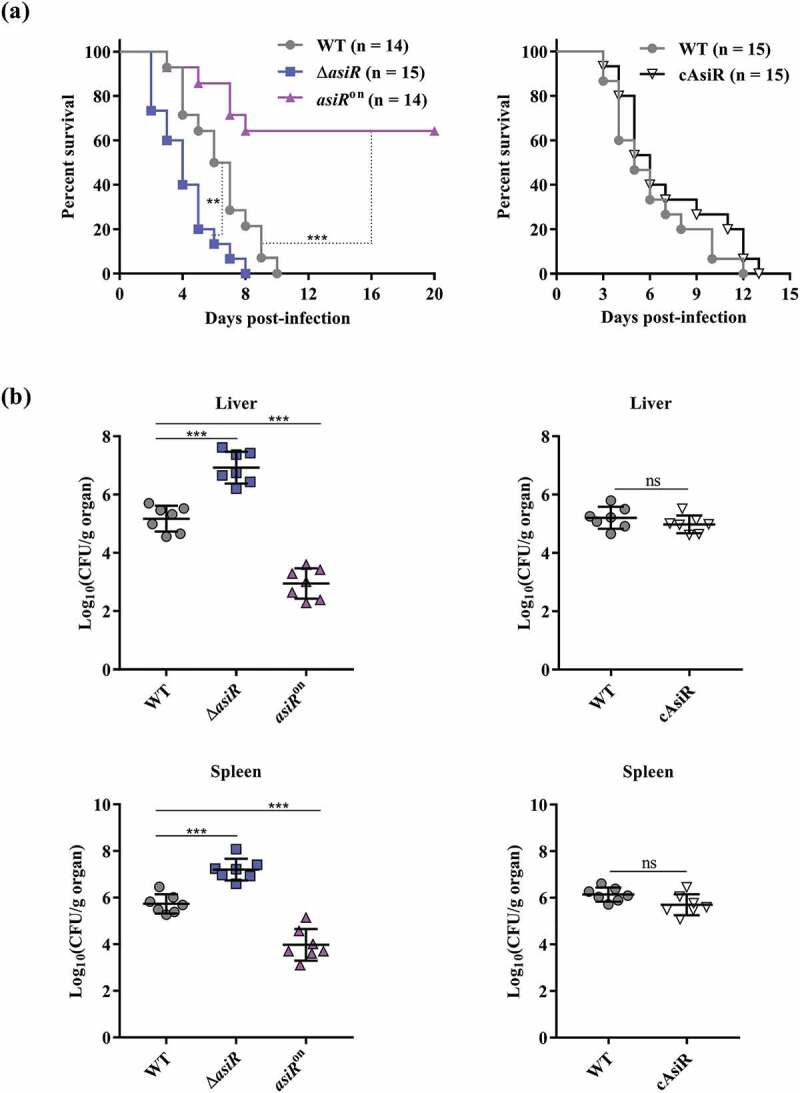
Deletion of *asiR* enhances *S*. Typhimurium virulence in mice. (a) Survival curves of mice infected intraperitoneally (i.p.) with the WT, Δ*asiR, asiR*^on^ or cAsiR. (b) Liver and spleen bacterial burdens in mice infected with WT, Δ*asiR, asiR*^on^ or cAsiR. Data were combined from two separate experiments and analyzed using Log-rank (Mantel-Cox) test (a) or Mann Whitney U-test (b). *P*-values: **, *P*< 0.01; ***, *P*< 0.001

Replication of *S*. Typhimurium within macrophages is essential for systemic infection. To validate the contribution of *asiR* downregulation in macrophages to *S*. Typhimurium virulence, mice were infected with WT, Δ*asiR, asiR*^on^, or cAsiR *via* i.p. injection and monitored for survival. All of the mice infected with Δ*asiR* died within 8 days with a mean survival time of 4 days, while the mice infected with WT and cAsiR died within 13 days with a mean survival time of 5 and 6 days, respectively (Figure 2(a)). The results indicate that the absence of *asiR* reduces the survival of infected mice. On the contrary, only 36% of the mice infected with *asiR*^on^ died within 8 days, with 64% surviving throughout the period of our experiment (20 days), indicating that the overexpression of *asiR* enhances the survival of infected mice. We further verified the virulence phenotype of WT, Δ*asiR, asiR*^on^, or cAsiR by determining their colonization abilities in the liver and spleen of infected mice. Three days after infection, Δ*asiR* showed significantly increased bacterial counts in the liver and spleen of infected mice compared to WT and reduced to the WT level by complementation with *asiR* (Figure 2(b)). The results indicate that the absence of *asiR* increases the colonization of *S*. Typhimurium in systemic loci. On the contrary, *asiR*^on^ showed decreased bacterial counts in the liver and spleen of infected mice compared to WT (Figure 2(b)), indicating that the overexpression of *asiR* reduces the colonization of *S*. Typhimurium in systemic loci. Therefore, the expression of *asiR* contributes negatively to *S*. Typhimurium virulence in mice, and it is downregulated in murine macrophages to promote *S*. Typhimurium replication and virulence.

### asiR contributes negatively to S. Typhimurium intracellular replication and virulence by positively regulating the expression of flagellar genes

Next, we investigated the mechanism underlying the negative contribution of AsiR to *S*. Typhimurium intracellular replication and virulence. SPI-2 genes are necessary for *S*. Typhimurium replication within macrophages and systemic infection in mice. Therefore, we tested whether AsiR influences the expression of SPI-2 genes. qRT-PCR analysis showed no significant change in the expression of three representative SPI-2 genes (*ssrA, ssaG*, and *sifA*) in Δ*asiR* compared to that in WT when bacteria were grown to stationary phase in N-minimal medium (Figure S3(a)) and during its growth inside RAW264.7 cells (Figure S3(b)), indicating SPI-2 gene expression is not affected by AsiR.

**Figure 3.**
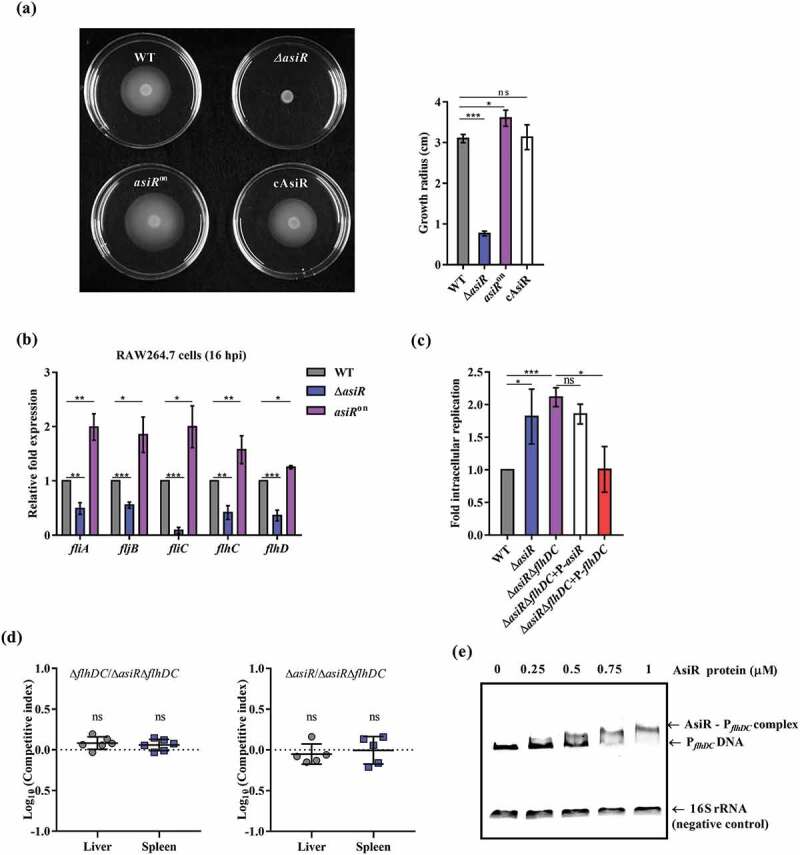
AsiR enhances bacterial motility and directly activates flagellar gene expression. (a) The representative images of swimming motility and growth radius after 8 h of incubation at 37°C on semi-solid LB agar medium of WT, Δ*asiR, asiR*^on^ or cAsiR. (b) qRT-PCR analysis of five flagellar genes (*fliA, fijB, fliC, flhC*, and *flhD*) expression in the WT, Δ*asiR*, or *asiR*^on^ that collected from infected RAW264.7 cells at 16 hpi. (c) Replication abilities of the WT, Δ*asiR*, Δ*asiR*Δ*flhDC*, Δ*asiR*Δ*flhDC+*p-*asiR* or Δ*asiR*Δ*flhDC+*p-*flhDC* in RAW264.7 cells. The fold replication was calculated as a ratio of the intracellular CFUs at 16hpi relative to that at 2 hpi. (d) Competitive index of the Δ*asiR* or Δ*flhDC* versus the Δ*asiR*Δ*flhDC* in the livers and spleens of i.p. infected mice. (e) EMSAs of *flhDC* promoter DNA fragments with increasing concentrations of the purified his_6_-AsiR protein. *16S rRNA* was used as a negative control. For (a, b, and c), data were obtained from three separate experiments and analyzed using Student’s t-test (a and c) or one-way ANOVA (b). For (d), data were combined from two separate experiments and analyzed using Mann Whitney U-test. *P*-values: *, *P*< 0.05; **, *P*< 0.01; ***, *P*< 0.001; ns, not significant

Through an *in vitro* phenotypic assay, the motility assay, we found that Δ*asiR* exhibited a defect in motility compared to WT and the defect was rescued by complementation with *asiR* (Figure 3(a)), implying that AsiR may regulate the expression of flagellar genes. We, therefore, tested the expression of representative flagellar genes including *fliC, fliA, fijB, flhC,* and *flhD* in WT, Δ*asiR, asiR*^on^, or cAsiR grown in LB and after infection of RAW264.7 cells for 16 h through qRT-PCR analysis. Under either condition, the expression of all the tested genes was downregulated in Δ*asiR* compared to that in WT, and restored to the WT level by complementation with *asiR* (Figure 3(b) and S4(a)), indicating that AsiR positively regulates the expression of flagellar genes. *asiR*^on^, which expresses *asiR* persistently in RAW264.7 cells (Figure 1(b)), showed enhanced motility (Figure 3(a)) and flagellar gene expression in LB and after infection of RAW264.7 cells (Figure 3(b) and S4(a)). Bioluminescent reporter assays further confirmed that the expression of the *flhDC* operon was decreased in Δ*asiR* while increased in *asiR*^on^ during bacterial growth within RAW264.7 cells (Figure S4(b)). Collectively, these results indicate that AsiR positively regulates the expression of flagellar genes.

The deletion of *asiR* in macrophages could result in the decrease of flagellar gene expression, leading to the enhanced intracellular replication of *S*. Typhimurium. To further verify that AsiR represses the intracellular replication and virulence of *S*. Typhimurium by activating flagellar gene expression. We next constructed *asiR/flhDC* double mutant strain (*∆asiR∆flhDC*) and two complemented strains (*∆asiR∆flhDC* strain complemented with *asiR* or *flhDC: ∆asiR∆flhDC+*P-*asiR, ∆asiR∆flhDC+*P-*flhDC*), and performed macrophage replication assay. The growth rate of *∆asiR∆flhDC* in RAW264.7 cells was comparable to that of *∆asiR*, indicating that mutation of *asiR* in ∆*flhDC* did not confer an additional replication advantage (Figure 3(c)). Meanwhile, the growth rate of *∆asiR∆flhDC+*P-*asiR* in RAW264.7 cells was comparable to that of *∆asiR∆flhDC* (Figure 3(c)), confirming that AsiR represses *S*. Typhimurium intracellular replication by activating flagellar gene expression.

We then conducted competitive infection assays to measure the difference in systemic tissue colonization between the single mutant strain (*∆flhDC* and *∆asiR*) and the double mutant (*∆asiR∆flhDC)*. The competitive ability of *∆asiR∆flhDC* was comparable with that of *∆flhDC* or *∆asiR* in both the liver and spleen of the infected mice (Figure 3(d)), indicating that mutation of *asiR* in ∆*flhDC* did not provide an additional virulence advantage. Collectively, these results suggest that AsiR contributes negatively to the intracellular replication and virulence of *S*. Typhimurium by activating the expression of flagellar genes.

### asiR positively regulates flagellar gene expression by directly binding to the promoter of flhDC

We next investigated whether AsiR regulates flagellar gene expression by binding to the *flhDC* promoter, which is a target of a broad range of transcriptional regulators that regulate the expression of flagellar genes[35]. The EMSA results showed that purified His-tagged AsiR shifted the *flhDC* promoter fragments in a concentration-dependent manner, but did not bind to the *16S rRNA* DNA (negative control) (Figure 3(e)). Therefore, AsiR directly binds to the *flhDC* promoter, leading to the activation of flagellar gene expression.

### The deletion of asiR in macrophages reduces the host inflammatory response

**Figure 4.**
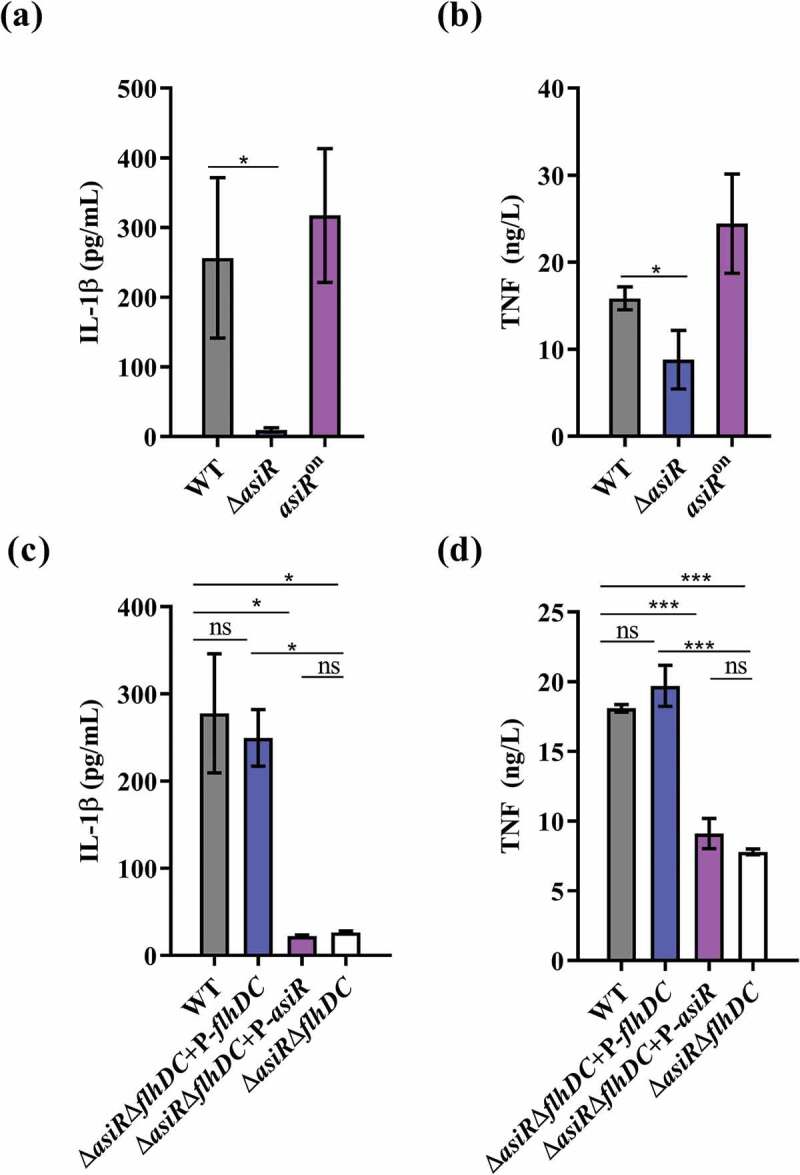
The repression of *asiR* leads to a reduction of pro-inflammatory cytokines secretion in macrophages. (a and b) The levels of Il-1β (a) and TNF-α (b) in the culture supernatants of RAW24.7 cells that infected WT, Δ*asiR*, or *asiR*^on^ for 16 h. (c and d) The levels of Il-1β (c) and TNF-α (d) in the supernatants of RAW24.7 cells infected with WT, Δ*asiR*Δ*flhDC+*p-*flhDC*, Δ*asiR*Δ*flhDC+*p-*asiR* or Δ*asiR*Δ*flhDC* for 16 h. Data were obtained from three separate experiments and analyzed using Student’s t-test. *P*-values: *, *P*< 0.05

As the flagellin triggers innate immune responses by stimulating the secretion of pro-inflammatory cytokines, such as IL-1β and TNF-α[36], we hypothesized that the deletion of *asiR* could reduce host inflammatory responses by reducing flagellin synthesis. To test this hypothesis, we infected RAW264.7 cells with WT, Δ*asiR*, or *asiR*^on^, and measured the amount of IL-1β and TNF-α in the culture supernatant at 16 hpi by ELISA. As shown in Figure 4(a, b), RAW264.7 cells infected with Δ*asiR* showed significantly decreased IL-1β and TNF-α levels than cells infected with WT or *asiR*^on^. The results indicate that the expression of *asiR* contributes positively to the secretion of pro-inflammatory cytokines. Further, the IL-1β and TNF-α levels in *∆asiR∆flhDC-*infected RAW264.7 cells were comparable to that of *∆asiR∆flhDC+*P-*asiR*-infected RAW264.7 cells, while the IL-1β and TNF-α levels in *∆asiR∆flhDC+*P-*flhDC-*infected RAW264.7 cells were comparable to that of WT-infected RAW264.7 cells (Figure 4(c, d)), indicating that AsiR promotes cellular inflammatory responses by activating flagellar gene expression. Therefore, *asiR* is downregulated to evade host detection by reducing flagellin synthesis and thereby reducing flagellin-stimulated inflammatory responses.

### The expression of asiR and therefore flagellar genes is downregulated in response to acidic pH in macrophages

**Figure 5.**
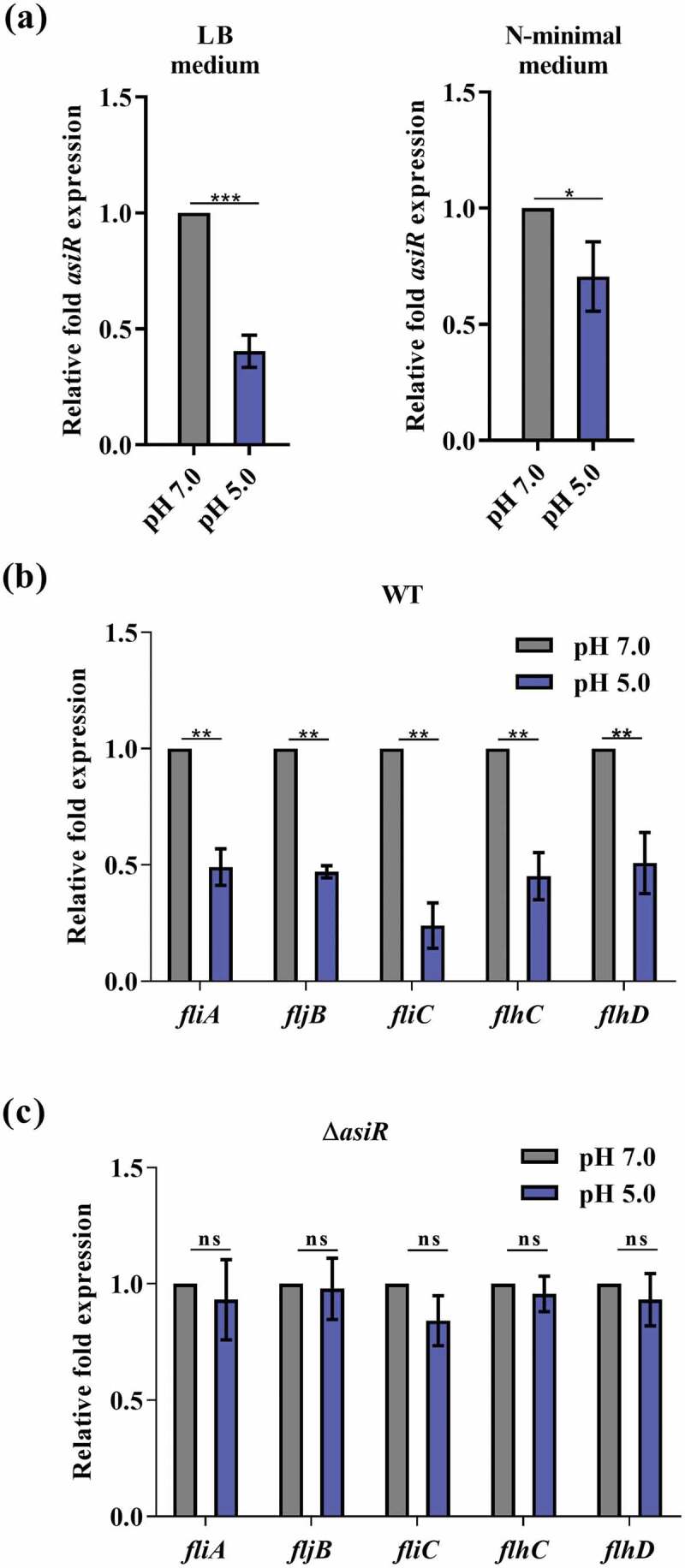
Acidic pH represses the expression of *asiR* and flagellar genes. (a) qRT-PCR analysis showed the expression levels of *asiR* in WT that grown in LB medium and N-minimal medium with pH 5.0 or pH 7.0. (b and c) qRT-PCR analysis showed the expression levels of *fliA, fijB, fliC, flhC*, and *flhD* in WT (b) and Δ*asiR* (c) that grown in N-minimal medium with pH 5.0 or pH 7.0. Data were obtained from three separate experiments and analyzed using Student’s t-test (a) or one-way ANOVA (b and c). *P*-values: *, *P*< 0.05; **, *P*< 0.01; ***, *P*< 0.001

To evaluate whether any of the known environmental cues in macrophages, including low Mg^2+^, low P_i_, low O_2_, and acidic pH, contribute to the downregulation of *asiR*, we examined the *asiR* expression of WT grown *in vitro* under defined conditions. According to qRT-PCR results, the expression of *asiR* was not significantly altered in the N-minimal medium with low Mg^2+^ & low P_i_ conditions compared to that in the medium with high Mg^2+^& high P_i_ conditions (Figure S5(a)). Moreover, the expression of *asiR* is not significantly altered under low O_2_ conditions compared to that under high O_2_ conditions (Figure S5(b)). However, the expression of *asiR* decreased significantly in WT grown in both LB and N-minimal medium with pH 5.0 compared to that in the medium with pH 7.0, as revealed by qRT-PCR analysis (Figure 5(a)) and confirmed by bioluminescent reporter assays (Figure S5(c)). These results indicated that the expression of *asiR* is repressed by mildly acidic pH.

Consistent with the downregulation of *asiR*, the expression of flagellar genes (*fliA, fijB, fliC, flhC,* and *flhD*) in WT was also downregulated in N-minimal medium at acidic pH (pH 5.0) (Figure 5(b)). In contrast, the expression of these flagellar genes was not changed at pH 5.0 in Δ*asiR* compared to that at pH 7.0 (Figure 5(c)) but downregulated in cAsiR (Figure S5(d)), indicating that *asiR* is required for the downregulation of flagellar genes at acidic pH. This was further confirmed by comparing the expression of flagellar genes in WT and Δ*asiR* at pH 5.0 and pH 7.0, respectively (Figure S5(e)). Therefore, the acidic conditions within macrophages can lead to the repression of *asiR* expression and its downstream flagellar genes.

## Discussion

**Figure 6.**
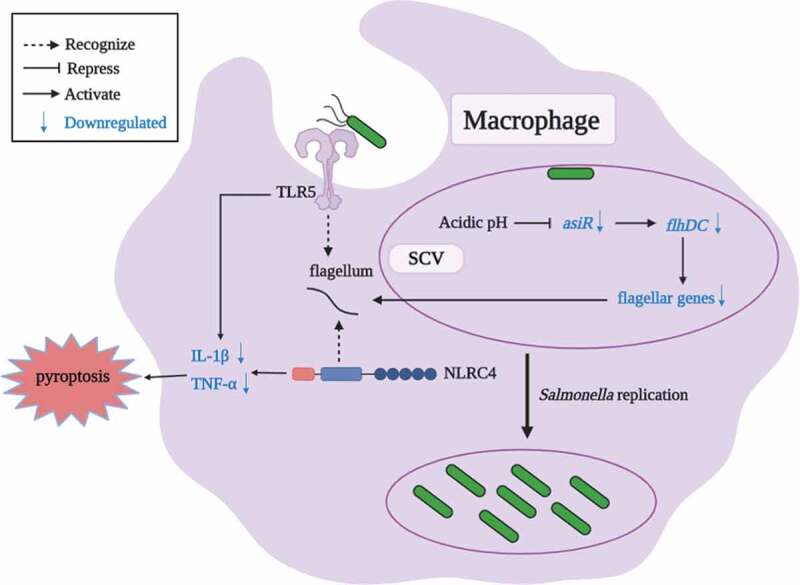
The acidic pH-mediated model of AsiR repressing flagellar gene expression in *S*. Typhimurium. After *S*. Typhimurium enters into macrophages, *asiR* expression is repressed in response to acidic pH within macrophages. AsiR binds to the promoter of *flhDC* that activates the expression of flagellar genes. The reduction in the expression of *asiR* gene leads to the downregulation of flagellar gene expression and pro-inflammatory cytokines secretion, contributing to the intracellular replication and systemic infection of *S*. Typhimurium

In this study, we revealed a novel regulatory mechanism mediated by AsiR for the downregulation of flagellar genes within host macrophages. AsiR positively regulates the expression of flagellar genes by binding to the *flhDC* promoter. The expression of *asiR* and therefore flagellar genes is downregulated within macrophages, and the acidic pH was identified as a host cue for this downregulation. The downregulation of flagellar genes mediated by AsiR contributes significantly to *S*. Typhimurium systemic pathogenesis, as indicated by the significant attenuated ability of *S*. Typhimurium to replicate in both macrophages and systemic organs when *asiR* was persistently expressed. We propose that *asiR* is downregulated in response to acidic pH signal of macrophages, followed by repression of the flagellar gene expression to increase the intracellular replication and pathogenicity of *S*. Typhimurium (Figure 6).

Flagellin is a ligand detected by the cell surface-localized Toll-like receptor 5 (TLR5) and also cytosolic NOD-like receptor 4 (NLRC4) [37,38]. The activation of the TLR5 signaling pathway induces the secretion of pro-inflammatory cytokines, including TNF-α[39]. The activation of the NLRC4 inflammasome elicits the caspase-1-dependent processes, which also promotes pro-inflammatory cytokines (e.g., IL-1β) production[40]. In agreement with the regulatory role of AsiR in flagellar gene expression, the production of IL-1β and TNF-α in the macrophages is significantly reduced due to the repression of AsiR. This result implicates that downregulation of flagellar gene expression by AsiR is an important mechanism utilized by *S*. Typhimurium to reduce the production of pro-inflammatory cytokines and replicate successfully within macrophages.

The assembly of flagella is regulated by complex mechanisms, including transcriptional, translational, and posttranslational regulation[35]. The *flhDC* flagellar master operon in *S*. Typhimurium activates transcription of other flagellar genes[41]. Several regulators have been reported to modulate flagellar gene expression by acting on FlhDC. The LysR-family protein HdfR represses flagellar gene expression through directly binding *flhDC* promoter to reduce *flhDC* transcription[42]. The global regulatory protein CsrA activates flagellar gene expression by protecting the translation of *flhDC* mRNA from RNase E-mediated cleavage[43]. STM1697 represses flagellar gene expression by binding to FlhD protein to repress the recruitment of RNA polymerase[25]. In this study, we found that AsiR activates *flhDC* and downstream flagellar gene expression at the transcriptional level by directly binding to the promoter of *flhD*C. In addition to promoting intracellular replication, AsiR may also contribute to *Salmonella* pathogenesis by affecting adherence and invasion, as flagella have been previously demonstrated to be required for the full adhesive and invasive potential of *S*. Typhimurium to intestinal epithelial cells [44,45]. Our preliminary investigation showed that Δ*asiR* exhibited a decreased ability to adhere to and invade HeLa cells (Figure S6). Whether AsiR has a physiological role in promoting adherence and invasion needs to be further investigated.

There are six potential transcriptional start-sites (TSSs) within the *flhDC* promoter region, to which many transcriptional regulators bind, including PhoP[46]. Several transcriptional regulators exert their control of *flhDC* expression at different stages of the bacterial growth phase through activating the transcription of two functional TSSs[46]. We demonstrate that AsiR binds directly to the *flhDC* promoter region. However, the exact AsiR binding sequence and its regulatory function in the growth phase are not identified in this study and will be the subject of future studies.

Inside the SCV of macrophages, the pH value has been estimated to be around 5[47]. It is known that SCV acidification promotes the intracellular replication of *S*. Typhimurium by inducing the expression of SPI-2 genes[48]. Although acidic pH can repress flagellar gene expression as revealed in minimal E glucose (minimal EG) medium[49], the underlying regulatory mechanisms are not clear. Here, we found that the expression of flagellar genes is downregulated in response to acidic pH via a novel regulator, AsiR, and confirmed that acidic pH also contributes to pathogenesis by repressing flagellar gene expression. Several regulatory systems are known to be responsive to the acidic signal, including OmpR/EnvZ and PhoP/Q [13,50]. Our preliminary investigation revealed that the deletion of *phoP* increased *asiR* expression in N-minimal medium at pH 5.0 based on qRT-PCR analysis, but the deletion of *ompR* did not influence *asiR* expression (Figure S7), suggesting that PhoP is a negative regulator of *asiR*. Whether PhoP/Q and/or other undiscovered systems are involved in acidic signaling-mediated downregulation of *asiR* and flagellar genes is a subject of future studies.

In summary, this work describes a novel reciprocal feedback between *S*. Typhimurium and host cells, in which AsiR responds to the acidic signal within macrophages and in return represses the immune reaction of host macrophages to promote bacterial intracellular replication.

## Supplementary Material

Supplemental Material
