# Supplementary material for: Downregulation of a novel flagellar synthesis regulator AsiR promotes intracellular replication and systemic pathogenicity of Salmonella Typhimurium
Source: Virulence. 2021 Jan 7;12(1):298–311. doi: 10.1080/21505594.2020.1870331 (PMC7808427; doi:10.1080/21505594.2020.1870331)
Supplement: Supplemental Material [file KVIR_A_1870331_SM5298.docx]

**Supplementary Materials**

**
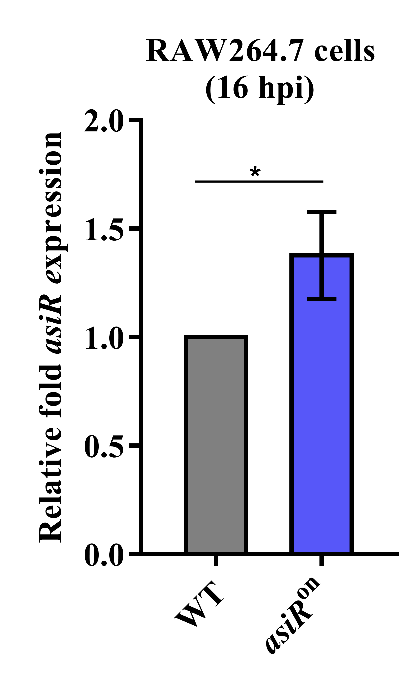
**

Figure S1. The high expression of *asiR* in *asiR* ^on^ during its growth in RAW264.7 cells. The RAW264.7 cells infected with WT or *asiR* ^on^ were lysed to collect the intracellular bacteria at 16 hpi. Fold change in *asiR* expression in *asiR* ^on^ relative to its expression in the WT was presented. Data were obtained from three separate experiments and analyzed using Student’s t-test. *P*-values: ^*^, *P* < 0.05.


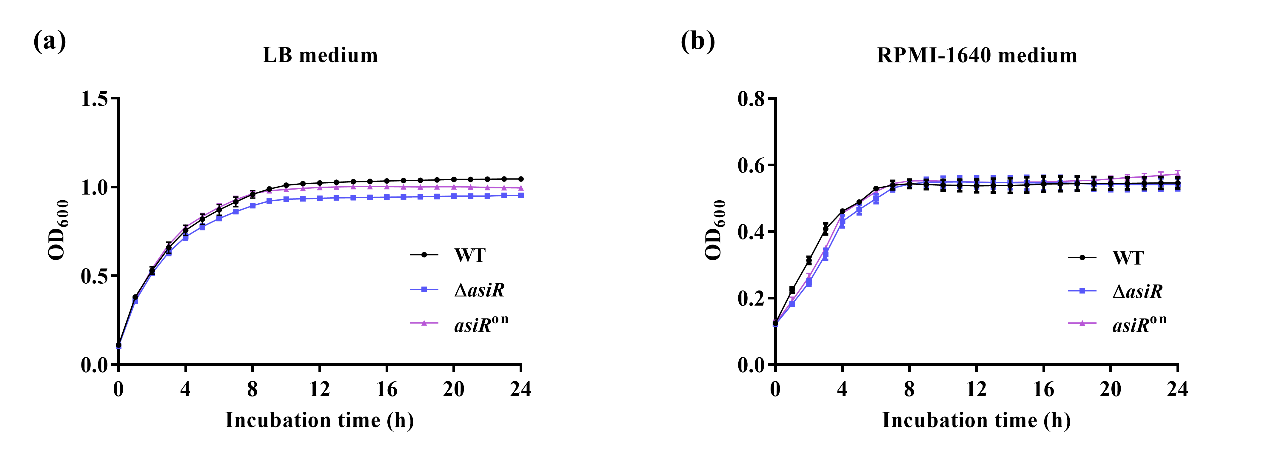


Figure S2. Mutation or overexpression of *asiR* did not affect the growth of *S.* Typhimurium in LB medium and RPMI‐1640 medium. (a and b) Growth curves of the WT, Δ*asiR*, and *asiR*^on^ in LB medium (a) and RPMI-1640 medium (b). Overnight cultures of WT, Δ*asiR*, and *asiR* ^on^ were diluted at 1:100 in fresh LB medium (a) and RPMI-1640 medium (b) and incubated in a shaking incubator (37 °C, 180 rpm) for 24 h. The measurements of optical density at 600 nm (OD_600_) of 1 mL bacterial culture were performed on a spectrophotometer every 1 h for 24 h. Data were obtained from three separate experiments.


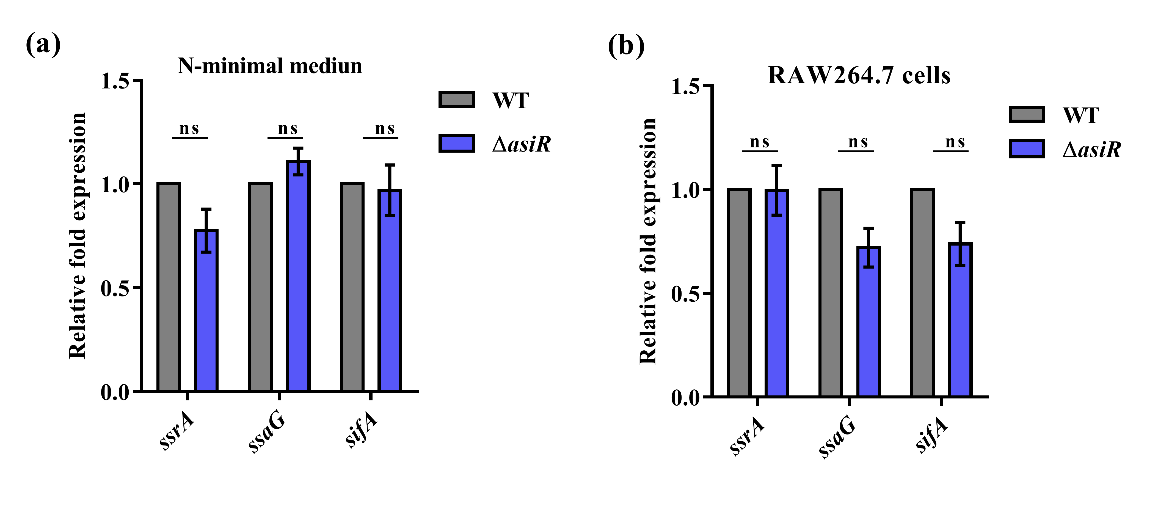


Figure S3. AsiR does not regulate SPI-2 gene expression. (a) qRT-PCR analysis of *ssrA*, *ssaG*, and *sifA* expression in the WT and Δ*asiR* that growth in N-minimal medium. (b) qRT-PCR analysis of *ssrA*, *ssaG*, and *sifA* expression in the WT and Δ*asiR* that collected from infected RAW264.7 cells at 16 hpi. Fold changes in their expression in Δ*asiR* relative to that in WT was presented. Data were obtained from three separate experiments and analyzed using one-way ANOVA. *P*-values: ns, not significant.


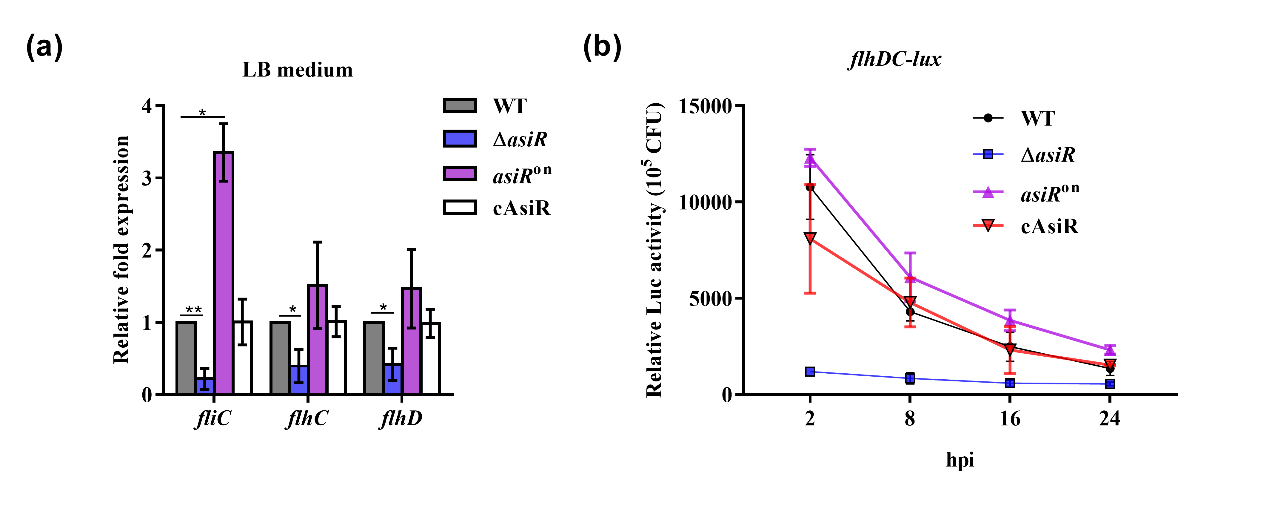


Figure S4. AsiR regulates positively the expression of flagellar genes. (a) qRT-PCR analysis of *fliC, flhC,* and *flhD* expression in the WT, Δ*asiR*, *asiR*^on^ or cAsiR that grown in LB medium. (b) Expression of the *flhDC-lux* transcriptional fusion was analyzed in the WT, Δ*asiR*, *asiR*^on^ or cAsiR inside RAW264.7 cells at 2, 8, 16, and 24 hpi. Data were obtained from three separate experiments and analyzed using one-way ANOVA. *P*-values: ^*^, *P* < 0.05; ^**^, *P*< 0.01; ^***^, *P*< 0.001; ns, not significant.


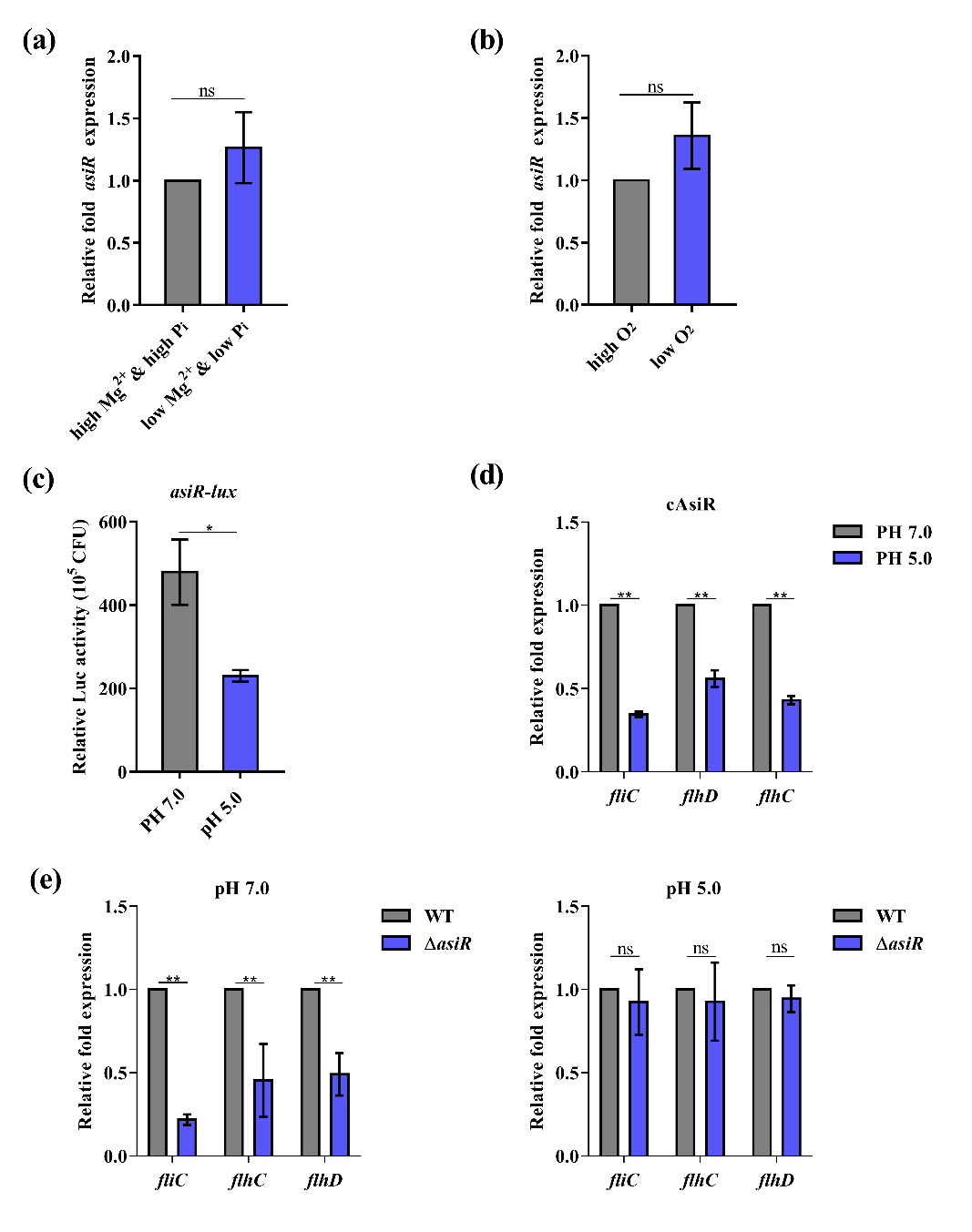


Figure S5. Acidic pH represses the expression of *asiR* and flagellar genes. (a) WT was cultured in N-minimal medium with low Mg^2+^ & low P_i_ conditions (10 μM Mg^2+^ and 1 μM P_i_) or with high Mg^2+^ & high P_i_ conditions (200 μM Mg^2+^ and 1 mM Pi) to the stationary phase. The expression level of *asiR* in N-minimal medium with low Mg^2+^ & low P_i_ conditions is presented as fold change relative to that in N-minimal medium with high Mg^2+^ & high P_i_ conditions. (b) WT was cultured in N-minimal medium under high O_2_ or low O_2_ conditions to the stationary phase. Fold change in *asiR* expression under low O_2_ relative to that under high O_2_ was presented. (c) Expression of the *asiR*-*lux* transcriptional fusion was analysed in WT that grown in N-minimal medium with pH 5.0 or pH 7.0. (d) qRT-PCR analysis of *fliC, flhC,* and *flhD* expression in cAsiR that grown in N-minimal medium with pH 5.0 or pH 7.0. (e) qRT-PCR analysis of *fliC, flhC,* and *flhD* expression in WT or Δ*asiR* that grown in N-minimal medium with pH 5.0 or pH 7.0. Data were obtained from three separate experiments and analyzed using Student’s t-test (a, b, and c) or one-way ANOVA (d and e). *P*-values: ^*^, *P* < 0.05; ^**^, *P*< 0.01; ^***^, *P*< 0.001; ns, not significant.


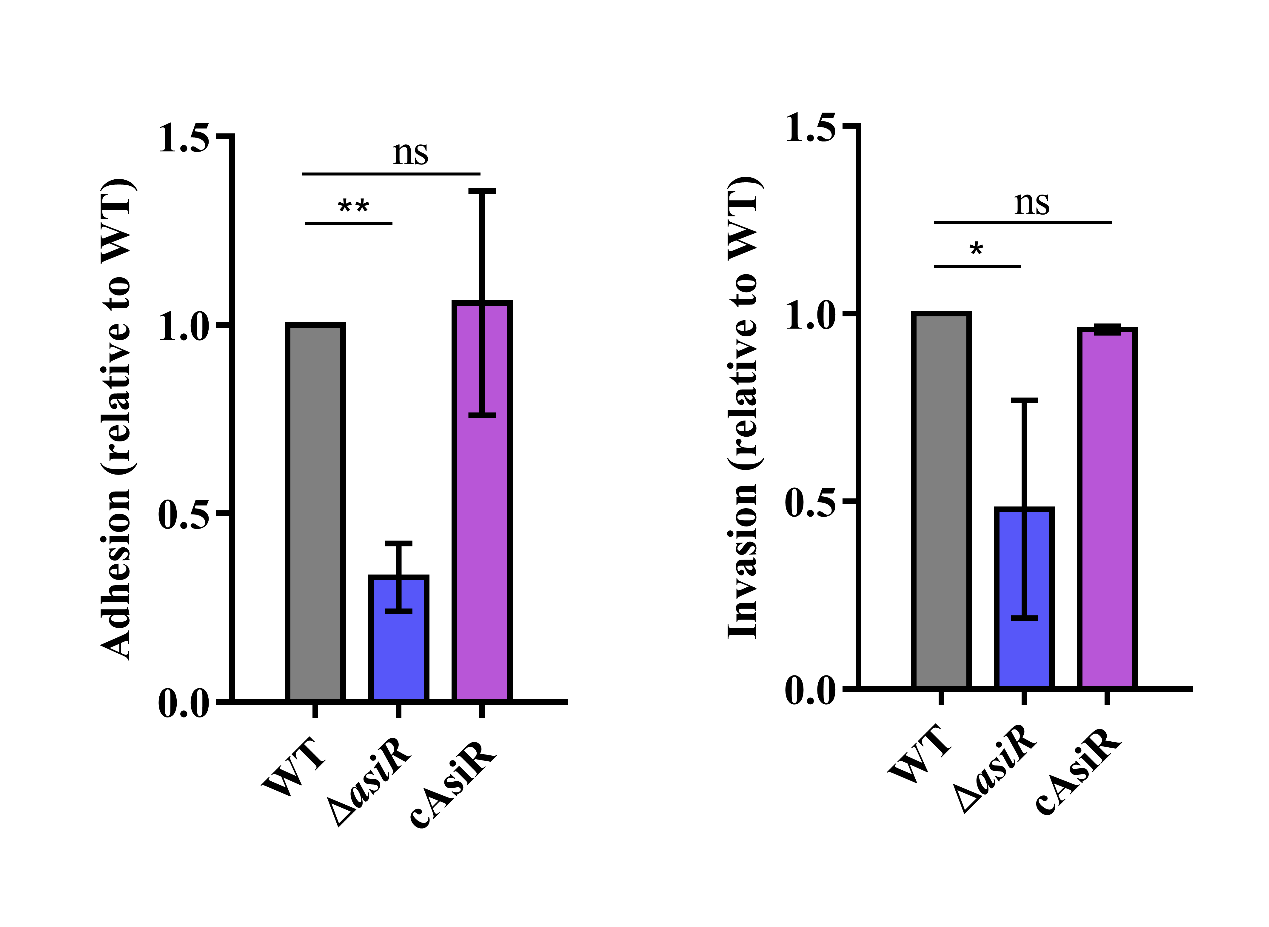


Figure S6. Deletion of *asiR* decreased *S.* Typhimurium adhesion to and invasion of Hela cells. Hela cells were infected with WT, Δ*asiR* or cAsiR at MOI of 50. Adhesion and invasion abilities of Δ*asiR* or cAsiR were calculated relative to that of WT. Data were obtained from three separate experiments and analyzed using Student’s t-test (c). *P*-values: ^*^, *P* < 0.05; ^**^, *P*< 0.01; ns, not significant.


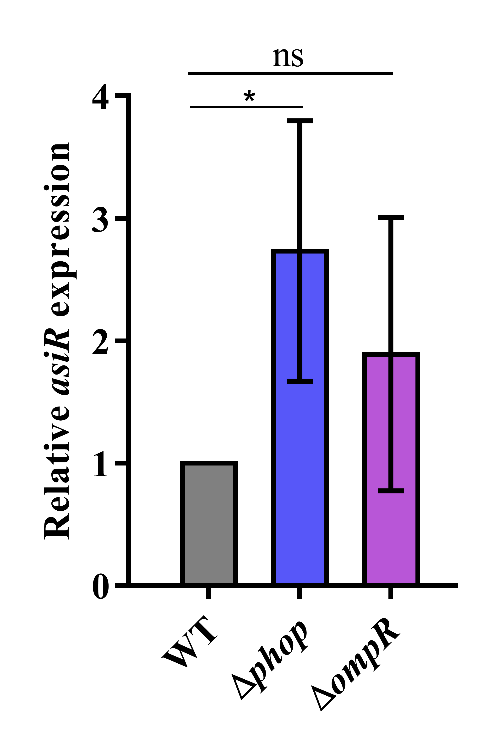


Figure S7. Deletion of *phoP* increased *asiR* expression. The WT, Δ*phop* or Δ*ompR* were grown to stationary phase in N-minimal medium with pH 5.0. The expression levels of *asiR* were determined by qRT-PCR*.* Fold changes in *asiR* expression in Δ*phop* or Δ*ompR* relative to that in WT was presented. Data were obtained from three separate experiments and analyzed using Student’s t-test. *P*-values: *, *P* < 0.05; ns, not significant.

**Table S1 Primers used in this study**

| **Target Gene** |  | **Primer Sequence (5′–3′)** |
| --- | --- | --- |
| **Primers for construction of strains** | | |
| Δ*asiR* | F | GCTTTGTGCCGCAATCAGCCCTCAAGAGCAGGAACTATGTGTAGGCTGGAGCTGCTTC |
|  | R | GGACAAAAAAGGAAAATGACGCCTGTACGTGAAGGGAACATATGAATATCCTCCTTAG |
| Δ*flhDC* | F | GTTTGTCCACACCGTTTCGGTTAAACAGCCTGTTCGATGTGTAGGCTGGAGCTGCTTC |
|  | R | GCGGCTACGTCGCACAAAAATAAAGTTGGTTATTCTGGCATATGAATATCCTCCTTAG |
| *ssrA-*promoter | F | GATTTTTCCGGGTTTAAGACGTGGGGGTTGAGGCTCCAT AATGCTTCCCTCCAGT |
|  | R | ATCGGCTATTACCTTCG |
| Cm | F | CGAAGGTAATAGCCGAT GTGTAGGCTGGAGCTGCTTC |
|  | R | CTGGATGAAAAACCTGAACCAAAGGTGACATTATGATTT CATATGAATATCCTCCTTAG |
| AsiR-his_6_ | F | CATGCCATGGATGGAGCCTCAACCCCCACGTC |
|  | R | CCCAAGCTTTTTTGTCATGTCCAGGGC |
| P-*asiR* | F | CGGGATCC AGCATCCAGCAGACTCAT |
|  | R | CGGAATTC CGCAGTCCAGTATCTCCA |
| P-*flhDC* | F | CGGGATCC CGCTGCTGGAGTGTTTGT |
|  | R | CGGAATTC GCTCATAACTCGCTCCTTG |
| P-*asiR-lux* | F | CCGCTCGAG CGCAGTCCAGTATCTCCA |
|  | R | CGCGGATCC TGTCACCTGTCTGGGGA |
| P-*ssrA-lux* | F | CCGCTCGAG ATCGGCTATTACCTTCG |
|  | R | CGCGGATCC TATGCTGCCGTTTCTG |
| P-*flhDC-lux* | F | CCGCTCGAG TGGGTGAACAAGGAAAGC |
|  | R | CGCGGATCC ATACCGAGGCGGAACATC |
| **Primers for identification of strains** | | |
| Δ*asiR* | F | GATGAAATGGTAGGGATTGTG |
|  | R | ACACCGTAAGAGCAACAGATT |
| *asiR*^on^ | F | CAATACGCTGGGAGGCACG |
|  | R | GGAGTGAATACCACGACGAT |
| Δ*flhDC* | F | AACCGCCGAAAACTGT |
|  | R | AGTGGGAGGCTGCGTT |
| **Primers for qRT-PCR** | | |
| *asiR* | F | GCCATTGAACTTGCCACTACC |
|  | R | CATCCACGACACCGAGAACC |
| *16s rRNA* | F | GAAAGCGTGGGGAGCAAAC |
|  | R | ACATGCTCCACCGCTTGTG |
| *ssrA* | F | CTGGACCTCTTGCTGGCTGAT |
|  | R | TGGCGTAAGTCGGTTAGTTCCT |
| *ssaG* | F | TGGATATGCTCTCCCACATGG |
|  | R | CTGCTGTAAGGCAAATTGCG |
| *sifA* | F | ATGATGCCACCATTATTCTTCG |
|  | R | CGTCATTTGTGGATGCGATT |
| *fliA* | F | CAAGGAACGGCATTTACCAC |
|  | R | CGATACTCCGCAACAGGGAT |
| *fijB* | F | AGGACAACACCCTGACCATCC |
|  | R | TTTCACATCATACGCTTTCTGC |
| *fliC* | F | TGGGTCTGGATACGCTGAATG |
|  | R | AACGGTAACTTTGGCGTAATAT |
| *flhC* | F | CCCGCAAGCAGAAGAAGGA |
|  | R | GCTGGTGAGCGTGGGTAATAA |
| *flhD* | F | ACCTCCGAGTTGCTGAAACAC |
|  | R | TTGCTGGAGATCGTCAACGC |
| **Primers for EMSAs** | | |
| *16s RNA* | F | GAAAGCGTGGGGAGCAAAC |
|  | R | ACATGCTCCACCGCTTGTG |
| *flhDC* promoter | F | AATGTGTTTTAGCAACTCGG |
|  | R | TGTTATCTATTATCCTGGCG |
